# Cholesterol Metabolism Is Altered in Rett Syndrome: A Study on Plasma and Primary Cultured Fibroblasts Derived from Patients

**DOI:** 10.1371/journal.pone.0104834

**Published:** 2014-08-12

**Authors:** Marco Segatto, Laura Trapani, Ilenia Di Tunno, Claudia Sticozzi, Giuseppe Valacchi, Joussef Hayek, Valentina Pallottini

**Affiliations:** 1 Dept. of Science, Section of Biomedical Science and Technologies, University of Roma Tre, Rome, Italy; 2 Dept. of Life Sciences and Biotechnology, University of Ferrara, Ferrara, Italy; 3 Child Neuropsychiatry Unit, University Hospital, Azienda Ospedaliera Universitaria Senese (AOUS), Siena, Italy; University of Insubria, Italy

## Abstract

Rett (RTT) syndrome is a severe neurological disorder that affects almost exclusively females. Several detectable mutations in the X-linked methyl-CpG-binding protein 2 gene (*MECP2*) are responsible for the onset of the disease. MeCP2 is a key transcription regulator involved in gene silencing *via* methylation-dependent remodeling of chromatin. Recent data highlight that lipid metabolism is perturbed in brains and livers of *MECP2*-null male mice. In addition, altered plasma lipid profile in RTT patients has been observed. Thus, the aim of the work is to investigate the protein network involved in cholesterol homeostasis maintenance on freshly isolated fibroblasts and plasma from both RTT and healthy donors. To this end, protein expression of 3-hydroxy-3methyl glutaryl Coenzyme A reductase (HMGR), sterol regulatory element binding proteins (SREBPs), low density lipoprotein receptor (LDLr) and scavenger receptor B-1 (SRB-1) was assessed in cultured skin fibroblasts from unaffected individuals and RTT patients. In addition, lipid profile and the abundance of proprotein convertase subtilisin/kexin type 9 (PCSK9) were analyzed on plasma samples. The obtained results demonstrate that the main proteins belonging to cholesterol regulatory network are altered in RTT female patients, providing the proof of principle that cholesterol metabolism may be taken into account as a new target for the treatment of specific features of RTT pathology.

## Introduction

Rett syndrome (RTT) is (OMIM ID: 312750) a severe neurological disorder that affects almost exclusively females, with a frequency of approximately 1∶10,000 live births [1]. This disorder was first recognized about 50 years ago by Andreas Rett [2]. In 1999 mutations in the gene *Methyl-CpG-binding protein 2* (*MECP2*) were identified as the cause of this pathology [3]. Several mutations in the X-linked *MECP2* gene are detectable, leading to wide genetical and phenotypical heterogeneity of the disease [4]. MeCP2, a key transcriptional regulator, is critically involved in gene silencing through methylation-dependent remodeling of chromatin structure [5], [6]. MECP2 is able to repress gene transcription through the association with different co-repressors [1], even though other research studies demonstrated an opposite effect in specific brain districts [7], [8]. Approximately 80% of RTT clinical cases show a typical clinical picture, characterized by loss of acquired cognitive, social, and motor skills in a typical four-stage neurological regression, together with development of autistic behavior [4].

RTT patients appeared to develop normally up to 6–18 months of age. Subsequently, they fall into developmental stagnation followed by rapid deterioration, loss of previously acquired speech, replacement of purposeful use of the hands with incessant stereotypies, which are characteristic of the syndrome [7]. Recent data have also demonstrated plasma lipid profile alterations in RTT patients [9]. Notably, imbalances in both high density lipoprotein (HDL) and low density lipoprotein (LDL) levels with respect to aged-matched healthy donors are present [9]. The perturbations in plasma lipid profile are accompanied by a dramatic reduction of Scavenger Receptor B1 (SRB1). SRB1 is ubiquitously expressed and plays pivotal roles cellular lipid uptake [9]. Among others, it mediates the uptake of HDL-derived cholesterol and cholesteryl ester in the liver and other tissues. Very recently, Buchovecky and colleagues (2013) [10] showed that cholesterol metabolism is perturbed in brains and livers of *Mecp2*-null male mice, and statins (inhibitors of cholesterol biosynthesis) ameliorate the systemic imbalance of lipid profile, alleviate motor symptoms and confer increased longevity in *Mecp2* mutant mice [10], suggesting that cholesterol homeostasis maintenance could be altered in patients affected by RTT. Despite this evidence, no specific data collected on human studies are present in literature about prospective alterations of cholesterol metabolism in RTT. Here, we investigated the protein network of cholesterol homeostasis maintenance in this rare disease. To this aim, freshly isolated human fibroblasts and plasma derived from both RTT and healthy donors were used as experimental models, and the protein levels of SRB1 [9], Sterol Regulatory Element Binding Proteins (SREBP), 3-Hydroxy-3-MethylGlutaryl-coenzyme A Reductase (HMGR) [11], Low Density Lipoprotein receptor (LDLr) [11], [12] were determined. Moreover, Proprotein Convertase Subtilisin/Kexin type 9 (PCSK9) expression [13] and lipid profile were estimated in plasma samples from RTT and healthy donors.

## Materials and Methods

### 2.1 Subjects and ethics statements

A total of 15 female patients with classical RTT syndrome (mean age: 20.3±12.3), and 15 healthy female controls of comparable age (mean age: 19.2±14.5) participated to the study. All the patients were consecutively admitted to the Child Neuropsychiatry Unit of the University Hospital of Siena (Head: J.H.). Patients were supplied with a Mediterranean diet. Olive oil was the main fat used for seasoning and for food preparation, whereas butter was completely devoid from the diet. In case of dysphagia, the same foods were mashed and administered to RTT individuals. None of the patients enrolled in this study was fed through a feeding tube. The dietary ratio among carbohydrates, proteins and fat was 50∶20∶30.

All the subjects were fasted before blood collection. Blood samplings in the control group were carried out during routine health, sport checks, or blood donations, while blood samplings in patients were obtained during the periodic clinical checks. The study was approved by the Institutional Review Board of University Hospital, Azienda Ospedaliera Universitaria Senese (AOUS), Siena, Italy and all informed consents were obtained in written form from either the parents or the legal tutors of the enrolled patients. This procedure is approved by the Institutional Review Board of University Hospital, Azienda Ospedaliera Universitaria Senese.

### 2.2 Determination of serum lipids levels

The subjects were fasted before blood sampling and plasma lipids were measured using specific diagnostic kits following manufacturer instructions. Serum concentration of total cholesterol, HDL-cholesterol and triglycerides, were performed on Cobas 6000 System (Roche Diagnostics, Italy). Total cholesterol was analyzed by an enzymatic method based on the determination of the Δ4-cholestenone following the enzymatic cleavage of cholesterol ester by cholesterol esterase, the transformation of cholesterol by cholesterol oxidase and the subsequent measurement of the hydrogen peroxide produced by using the Trinder reaction [14]. HDL-cholesterol was estimated with a homogeneous enzymatic colorimetric assay with cholesterol esterase, cholesterol oxidase, and 4-aminoantipyrine [15]. Triglycerides were measured by an enzymatic colorimetric method based on the oxidation of glycerol (obtained by triglyceride hydrolysis), to dihydroxyacetone phosphate and hydrogen peroxide. These compounds, together with 4-aminophenazone and 4-chlorophenol, form a colored product by peroxidase reaction.

### 2.3 Cells

Human skin fibroblasts were isolated from 3-mm skin punch biopsy (n = 15 for RTT and n = 15 for controls). Cells were cultured in DMEM (Sigma-Aldrich, Milan, Italy), containing 20% fetal calf serum [9] (FCS, Sigma-Aldrich) and antibiotics (100 U/ml penicillin, 100 µg/ml streptomycin) (Lonza, Milan, Italy). Cells were incubated at 37 C° in a humidified atmosphere at 5% CO2 for 3 days. When fibroblasts growing from the dermal pieces formed a confluent layer, the dermal pieces were removed and trypsin/EDTA mixture (Sigma-Aldrich) was added to separate fibroblasts. Cells were transferred to 25-cm^2^ culture flasks (Falcon, Perugia, Italy) and subcultured in 10% FCS/DMEM. Fibroblasts from passage 3 to 5 were used for the experiments. 1×10^6^ cells were seeded in each flask (25 cm^2^), whereas the experiments were performed when cells reached 70/80% confluence.

### 2.4 Lysate preparation, electrophoresis, and protein level analysis

Cells were lysed in 80 µl lysis buffer (0.25 M Tris pH 6.8, 10% SDS, 0.062 M NaF, and protease inhibitors) by sonication (20 sec pulse sonication, 40% intensity). Plasma was diluted 1∶20 in the lysis buffer. Protein concentration of each sample was determined by the method of Lowry [16]. Protein profiles were analyzed by Western blotting following the previously used protocol [17]. Briefly, 30 µg of protein from cell lysates were resolved by 7% SDS-PAGE at 120 V for 60 min. The proteins were subsequently transferred electrophoretically onto nitrocellulose membrane for 90 min at 100 V. The nitrocellulose membrane was blocked at room temperature with 5% fat-free milk in Tris-buffered saline (138 mM NaCl, 27 mM KCl, 25 mM Tris-HCl, 0.05% Tween-20, pH 6.8), and probed at 4°C overnight with primary antibodies against HMGR (1∶1000 code: 07-457 Upstate, Lake Placid, NY; host: rabbit); LDLr (1∶1000 code: ab30532 Abcam, Cambridge, UK; host: rabbit); SRB1 (1∶1000 code: H-180 Santa Cruz Biotechnology, Heidelberg, Germany; host: rabbit); PCSK9 (1∶1000 code: H-160 Santa Cruz Biotechnology, Heidelberg, Germany; host: rabbit); SREBP-1 (1∶1000 code: ab135133 Abcam, Cambridge, UK; host: rabbit) and SREBP-2 N-terminal (1∶1000 code: ab28481 Abcam, Cambridge, UK; host: rabbit) which recognize the nuclear cleaved and transcriptionally active fragment [18]. The first incubation was followed by another 1 h incubation at room temperature with secondary anti-rabbit IgG antibody coupled to horseradish peroxidase (1∶10000 code: 170-6515 Bio-Rad Laboratories, Milan, Italy; host: goat). The nitrocellulose membrane was then stripped with Restore Western Blot Stripping Buffer (Pierce Chem-ical, Rockford, IL, USA) for 10 min at room temperature and re-probed with anti-α-tubulin (MP Biomedicals) antibody. Thus, each reported value was derived from the ratio between arbitrary units obtained by the protein band of interest and the respective tubulin (chosen as housekeeping protein) or Ponceau S staining. Bound antibodies were visualized using enhanced chemoluminescence detection (GE Healthcare). All images derived from Western blotting were analyzed with ImageJ (NIH, Bethesda, MD, USA) software for Windows.

### 2.5 HMGR activity assay

The assay was performed by a radioisotopic method, following the production of C^14^MVA (mevalonate) from 3-[^14^C]-HMGCoA (specific activity 57.0 mCi/mmol. Amersham-Pharmacia, Little Chalfont, UK). Cells were homogenized in phosphate buffer containing 0.1 M sucrose, 0.05 M KCl, 0.04 M KH_2_PO_4_, 0.03 M EDTA, 50 µM NaF, pH 7.4. Hundred micrograms of proteins were incubated in presence of co-factors (20 mM glucose-6-phosphate, 20 mM NADP sodium salt, 1 unit of glucose-6-phosphate dehydrogenase, and 5 mM dithiothreitol). Final volume was 180 µl. The assay was started by the addition of 10 µl (0.088 μ Ci/11.7 nmol) of 3-[^14^C]-HMG-CoA. The radioactivity of the synthesized [^14^C]-MVA, isolated by chromatography on AG1-X8 ion exchange resin (BioRad, Italy), was measured, and the recovery was calculated on the basis of the internal standard (3-[^3^H]-MVA, specific activity 24.0 Ci/mmol. Amersham-Pharmacia, Little Chalfont, UK) [17].

### 2.6 Statistics

Data were analyzed with unpaired Student's t test. Values of p<0.05 were considered statistically significant. Statistical analysis was performed using GRAPHPAD INSTAT3 (GraphPad, La Jolla, CA, USA) for Windows.

## Results and Discussion

**Figure 1:**
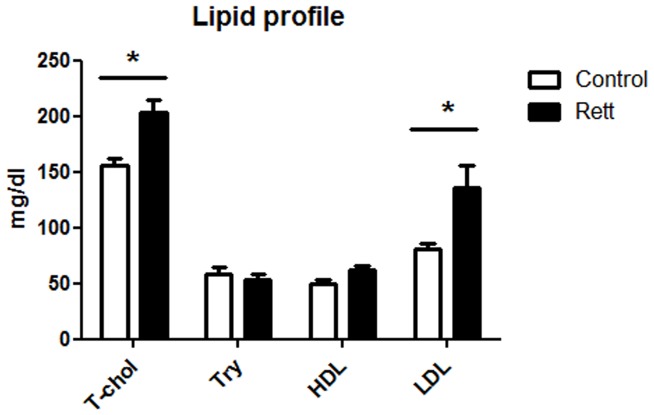
Plasma lipid profile in Rett and healthy donors. The figures shows the levels of total cholesterol (T-chol), triglycerides (Try), high density lipoproteins (HDL), and low density lipoproteins (LDL) in Rett patients and in healthy donors. Data are the mean ± S.D. of n = 3 independent experiments carried out on 15 subject for each experimental group. Data were analyzed with unpaired Student's t test. * = p<0.05.

**Figure 2:**
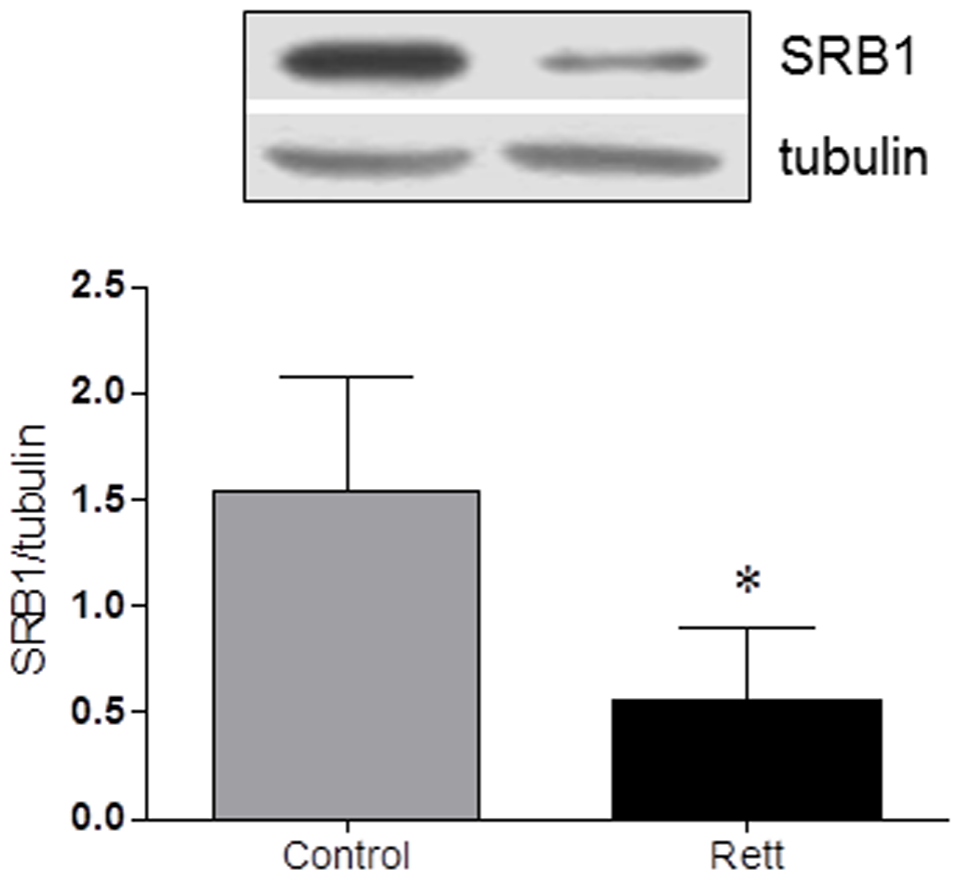
SRB1 protein levels in Rett- and healthy donor-derived fibroblasts. The figure shows a representative Western blot and the densitometric analysis of SRB1 protein levels. The protein levels were normalized with tubulin content. The data are expressed as arbitrary units obtained analyzing the bands by using the software ImageJ. Data are the mean ± S.D. of n = 3 independent experiments carried out on 15 subject for each experimental group. Data were analyzed with unpaired Student's t test. * = p<0.05.

The aim of the presented work was to study the protein network of cholesterol homeostasis maintenance in RTT. To this purpose, biochemical analysis were performed on freshly isolated fibroblasts and plasma from patients and healthy donors. The subjects enrolled for this study were checked for plasma lipid profile. Figure 1 demonstrates that total cholesterol and LDL-cholesterol are significantly higher in RTT patients than in healthy controls. This finding is in accordance with a recent published work, which highlighted that LDL concentration is significantly elevated in *Mecp2*-null mice [10]. HDL content is also shown to be increased in RTT, further supporting other recent and published data [9]. However in this case, the difference is not statistically significant: the discrepancy in the statistical significance could be related to a smaller group of subjects who participated in this work if compared with the number of persons enrolled in the study performed by Sticozzi and colleagues [9]. On the contrary, no variations are observable in triglycerides content. Protein level expression of SRB1 in primary fibroblasts from RTT and healthy donors was also checked. Western blot analysis highlights that SRB1 expression is lower (∼70%) in RTT cells than in healthy fibroblasts (Figure 2), confirming the previously obtained results [9].

Once ascertained the reliability of the experimental model of fibroblast culture and the prospective deregulations both in plasma cholesterol profile and in SRB1, the main proteins involved in the regulatory network of cholesterol metabolism were evaluated. To start, the attention was focused on HMGR, the rate and limiting enzyme of cholesterol biosynthetic pathway [19], [20].

**Figure 3:**
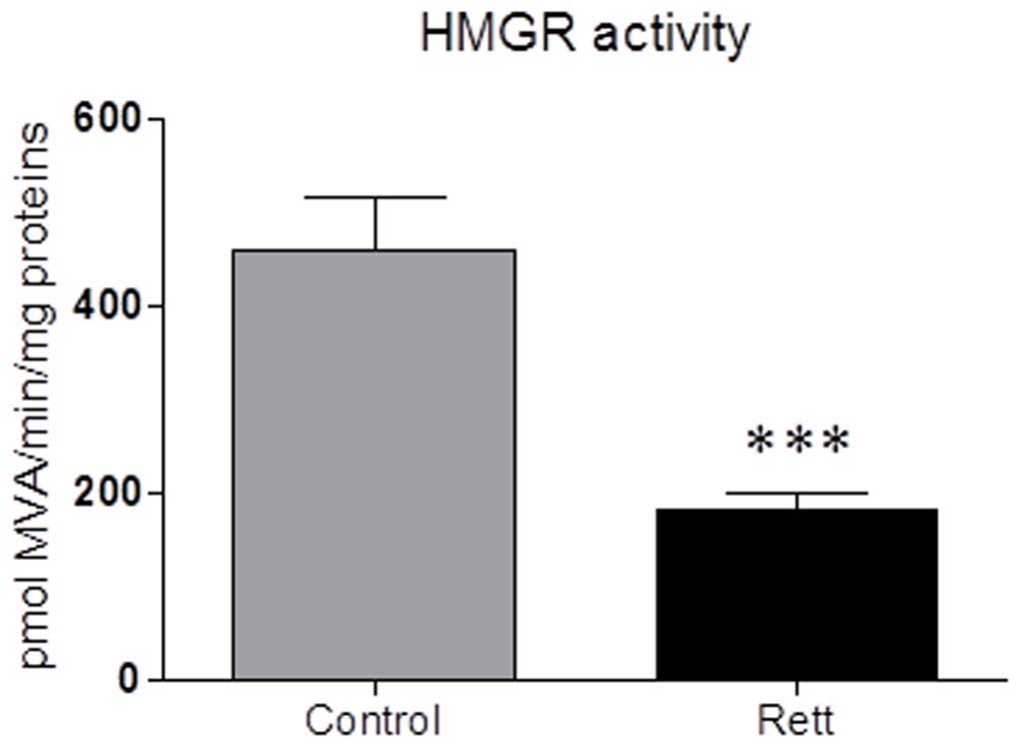
HMGR activity in Rett- and healthy donor-derived fibroblasts. The figure illustrates HMGR activity performed in Rett- and healthy donor-derived fibroblasts. The activity of the enzyme is expressed as [14C]-mevalonate production (pmol/min/mg proteins) from 3-[14C]-HMG CoA added to the samples. For details see the main text. Data are the mean ± S.D. of n = 3 independent experiments carried out on 10 subject for each experimental group. Data were analyzed with unpaired Student's t test. *** = p<0.001.

**Figure 4:**
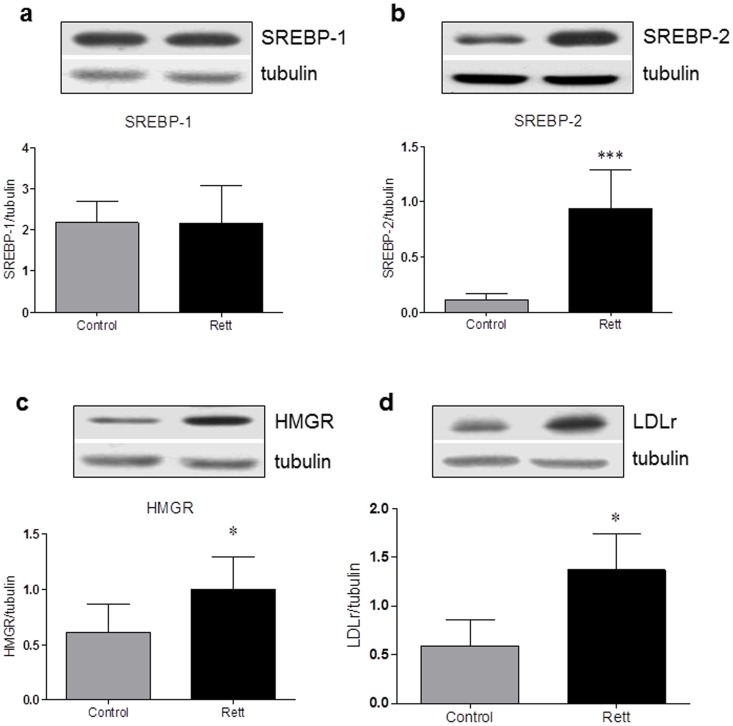
SREBP-1, SREBP-2, HMGR, and LDLr protein levels in Rett- and healthy donor-derived fibroblasts. The figure shows a representative Western blot and the densitometric analysis of SREBP-1 (a), SREBP-2 (b), HMGR (c), and LDLr (d) protein levels. The protein levels were normalized with tubulin content. The data are expressed as arbitrary units obtained analyzing the bands by using the software ImageJ. Data are the mean ± S.D. of n = 3 independent experiments carried out on 15 subject for each experimental group. Data were analyzed with unpaired Student's t test. * = p<0.05 *** = p<0.001.

HMGR activity undergoes short-term regulation operated by phosphorylation/dephosphorylation mechanisms which respectively inhibit and activate the enzyme through the activity of AMP activated kinase and Protein Phospatase 2 A [21], [22]. The enzymatic assay was performed in presence of NaF that preserves the *in vivo* phosphorylation status by permanently blocking the activity of NaF-sensitive phosphatases [23]. The enzyme activity results to be lower (170.5±5.48 pmol MVA produced/min/mg prot) in RTT-derived fibroblasts than in healthy donors (460.0±34.06 pmol MVA produced/min/mg prot) (Figure 3), suggesting that cholesterol synthesis is reduced in RTT fibroblasts. The suppression in HMGR activity strongly corroborates with the results obtained from Buchovecky and collaborators, who reported a strong decrease in sterol synthesis in the adult brains of *Mecp2* mutant mice [10]. Thus, we evaluated the amount of the transcription factors SREBPs, which are modulated in dependence on the intracellular sterol content. Notably, when the amount of sterolic compounds is low, SREBPs are proteolytically processed, the transcriptionally active fragments are released from the endoplasmic reticulum (ER), migrate into the nucleus and activate the transcription of target genes involved in the regulation of lipid metabolism (e.g. HMGR, LDLr); on the contrary, when sterols build-up into the cell, SREBPs are retained into the ER, thus leading to a decrease in gene transcription [24]. The amount of the nuclear and transcriptionally active fragment of SREBP-2 (Figure 4, panel b) is increased in RTT fibroblasts (∼90% fold increase), supporting the hypothesis that alterations in cholesterol content occurs in RTT cells. As a matter of fact, SREBP-2 is mainly committed to the transcription of genes whose products are involved in cholesterol metabolism [25]. Moreover, no changes are detectable in the protein expression of SREBP-1 transcriptionally active fragment between the two experimental groups (figure 4 panel a). As SREBP-1 is mainly committed to the transcriptional regulation of genes involved in fatty acid and triglyceride metabolism [25], these data corroborates with the lack of any change in the amount of plasma triglycerides observed in RTT patients. Thus, the present findings indicate that specific imbalance in cholesterol metabolism, rather than in the homeostasis of other lipids, are present in RTT.

The increased levels of the nuclear and transcriptionally active fragment of SREBP-2 prompt us to hypothesize that different products of key target genes involved in cholesterol metabolism could be up-regulated in RTT cells. Since cholesterol homeostasis is guaranteed by a fragile equilibrium between biosynthesis and uptake, HMGR and LDLr protein expression were analyzed. LDLr is a glycoprotein receptor able to regulate plasma cholesterol levels by removing LDL from the bloodstream [26] and, together with HMGR, represents one of the most sensitive targets for SREBP-2-dependent transcription [25], [27], [28]. As expected, both HMGR and LDLr protein levels are increased in RTT fibroblast if compared to healthy donor ones (∼35% and ∼60% respectively) (figure 4 panel c and d).

**Figure 5:**
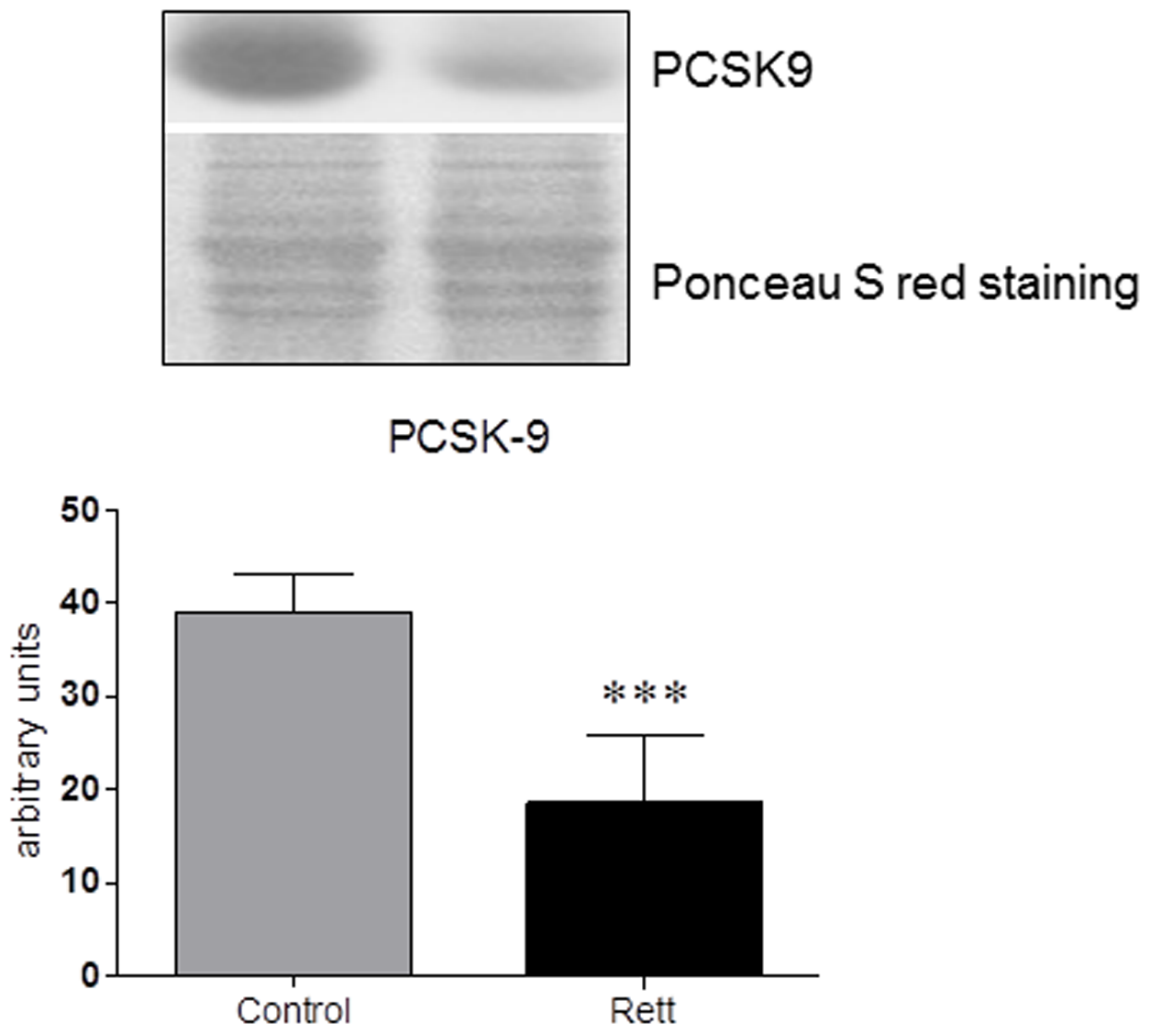
Plasma PCSK9 protein levels in Rett patients and healthy donors. The figure shows a representative Western blot and the densitometric analysis of PCSK9 protein levels. The samples were normalized for protein loading by using Ponceau S staining. The data are expressed as arbitrary units obtained analyzing the bands by using the software ImageJ. Data are the mean ± S.D. of n = 3 independent experiments carried out on 15 subject for each experimental group. Data were analyzed with unpaired Student's t test. *** = p<0.001.

Besides transcriptional events, LDLr protein levels are also modulated by PCSK9, a 72 kDa protease highly expressed in the liver and secreted into the systemic circulation. The binding of PCSK9 to LDLr results in the redistribution of LDLr from the cell membrane to lysosomes. PCSK9 seems to modify the itinerary of LDLr, redirecting the internalized LDLr to lysosomes for degradation and preventing its recycling to the plasma membrane [29]. Thus, the amount of PCSK9 was checked in plasma samples collected from RTT and healthy donors. The results demonstrated that PCSK9 plasma levels are markedly reduced (∼50%) in RTT patients (Figure 5): the lower PCSK9 plasma content could account, together with the increase in cellular SREBP-2 active fraction, for the high LDLr levels found in RTT fibroblasts, suggesting that both transcriptional and degradation pathways could be responsible for the alteration observed in the protein expression of this lipoprotein receptor.

Taken together, the present results suggest that the protein network of cholesterol homeostasis maintenance is deeply altered in RTT. Notably, the low intracellular cholesterol biosynthesis, as a consequence of the reduced HMGR activity, induces a classical feedback response, which determines the increase of the transcriptionally active SREBP-2 and the consistent rise in LDLr and HMGR protein content. In addition, the increase in LDLr is further supported by the reduction of PCSK9 plasma levels. It is interesting to note that LDLr overexpression in RTT fibroblasts is not able to counteract the abnormal elevation of plasma cholesterol content. This phenomenon could depend on several factors, which require further investigations. For instance, defects in LDLr membrane exposure, endocytosis and intracellular processing,or increase of dietary cholesterol absorption from the intestine, could represent valuable hypothesis to explain the discrepancy between LDL receptor expression and plasma cholesterol profile. In addition, as RTT is associated with somatic mosaicism for MECP2 [30],the authors cannot exclude that other organs/cells expressing wild-type MECP2could attempt to compensate the reduction of HMGR activity in MECP2-mutated cells with cholesterol and/or lipoprotein overproduction.

Even though this work highlights deep alterations in the main proteins involved in cholesterol homeostasis maintenance, the molecular mechanisms underlying the differences in cholesterol metabolism between RTT patients and healthy donors are still unknown and, to date, it is difficult to predict how cholesterol imbalance could affect brain development and neuronal functions in RTT individuals. Notably, the isolation of brain cholesterol from the whole body, the presence of redundant homeostatic mechanisms and the intercellular cholesterol shuttling in the central nervous system [31] render more intricate the causality linking cholesterol metabolism and RTT pathology.

A well-accepted hypothetical model for cholesterol homeostasis in the brain suggests that during the embryonic stage, before astrocyte differentiation, neurons are able to face their cholesterol need by biosynthesis. Postnatally, neurons reduce or even abandon their own synthesis and import cholesterol from astrocytes that produce and extrude cholesterol mainly through ATP Binding Cassette A1 (ABCA1) [31].Thus, the idea that neurons depend constitutively on astrocyte-derived cholesterol postulates that any interference with cholesterol delivery leads to neurological disorders [32]. Cholesterol shuttle involves several steps that may be perturbed in injuries or diseases. Experimental evidence collected by Buchovecky and collaborators strengthen this hypothesis, as modulators of cholesterol biosynthesis alleviate motor symptoms and conferred increased longevity in *Mecp2* mutant mice [10]. From these observations, we speculate that cholesterol biosynthesis, intercellular transport and/or uptake between neurons and astrocytes could be impaired in RTT individuals, leading to neurodevelopmental aberration.

In conclusion, our data demonstrated that the protein network involved in cholesterol metabolism is altered not only in *Mecp2* mutant mice, but also in fibroblasts derived from RTT female patients, thus providing the proof of principle that proteins and enzymes belonging to the regulatory machinery of cholesterol metabolism may be considered as new therapeutic targets for treating specific clinical features of RTT as already demonstrated in rodents [1], [10], [33].
