# Correction: Cholesterol Metabolism Is Altered in Rett Syndrome: A Study on Plasma and Primary Cultured Fibroblasts Derived from Patients

**DOI:** 10.1371/journal.pone.0114654

**Published:** 2014-11-24

**Authors:** The PLOS ONE Staff

There is an error in the funding statement. Please refer to the correct funding statement below.

The present research project funded by the Tuscany Region (Contract Health 2009 project no. TR142, Italy). The funders had no role in study design, data collection and analysis, decision to publish, or preparation of the manuscript.
